# Balancing Evidence and Uncertainty when Considering Rubella Vaccine Introduction

**DOI:** 10.1371/journal.pone.0067639

**Published:** 2013-07-05

**Authors:** Justin Lessler, C. Jessica E. Metcalf

**Affiliations:** 1 Department of Epidemiology, Johns Hopkins Bloomberg School of Public Health, Baltimore, Maryland, United States of America; 2 Department of Zoology, Oxford University, Oxford, United Kingdom; National Institutes of Health, United States of America

## Abstract

**Background:**

Despite a safe and effective vaccine, rubella vaccination programs with inadequate coverage can raise the average age of rubella infection; thereby increasing rubella cases among pregnant women and the resulting congenital rubella syndrome (CRS) in their newborns. The vaccination coverage necessary to reduce CRS depends on the birthrate in a country and the reproductive number, R_0_, a measure of how efficiently a disease transmits. While the birthrate within a country can be known with some accuracy, R_0_ varies between settings and can be difficult to measure. Here we aim to provide guidance on the safe introduction of rubella vaccine into countries in the face of substantial uncertainty in R_0_.

**Methods:**

We estimated the distribution of R_0_ in African countries based on the age distribution of rubella infection using Bayesian hierarchical models. We developed an age specific model of rubella transmission to predict the level of R_0_ that would result in an increase in CRS burden for specific birth rates and coverage levels. Combining these results, we summarize the safety of introducing rubella vaccine across demographic and coverage contexts.

**Findings:**

The median R_0_ of rubella in the African region is 5.2, with 90% of countries expected to have an R_0_ between 4.0 and 6.7. Overall, we predict that countries maintaining routine vaccination coverage of 80% or higher are can be confident in seeing a reduction in CRS over a 30 year time horizon.

**Conclusions:**

Under realistic assumptions about human contact, our results suggest that even in low birth rate settings high vaccine coverage must be maintained to avoid an increase in CRS. These results lend further support to the WHO recommendation that countries reach 80% coverage for measles vaccine before introducing rubella vaccination, and highlight the importance of maintaining high levels of vaccination coverage once the vaccine is introduced.

## Introduction

In vaccination policy, rubella is an unusual case because introduction of a safe and effective vaccine can lead to an increase in severe disease. This is because the most severe outcome of rubella infection, congenital rubella syndrome (CRS), occurs in the newborns of pregnant women infected in the first trimester of pregnancy. When a vaccine to a disease is introduced at levels insufficient to eliminate the disease, the result may be to increase the average age of infection. For many diseases this is a good thing, because older children and adults tend to experience less severe outcomes than young children. However, for rubella, an increase in the average age of infection may lead to an increased risk of rubella among women of child bearing age, and hence an increase in CRS. Caution in the introduction of rubella-containing vaccine is supported by observations of suspected vaccination-associated transient increases in the CRS burden in Greece and Costa Rica [1], [2].

Epidemic theory and empirical observation show that the average age of infection for a vaccine preventable disease conferring lifelong immunity is predominantly determined by the population birthrate, the level of vaccine coverage and the transmissibility of the disease. In most populations the birthrate is known to some degree of accuracy through census data. Because rubella vaccine is most often distributed as part of a bivalent measles-rubella (MR) vaccine or trivalent measles-mumps-rubella (MMR) vaccine, the coverage that will be obtained upon introduction of rubella vaccine is known with reasonable certainty based on current measles vaccine coverage. However, transmissibility, generally characterized by the basic reproductive number, R_0_, is not so easily measured. R_0_ is defined as the number of individuals a single infectious individual is expected to infect in a fully susceptible population, and is a result of biological, environmental and social factors. Because R_0_ depends on factors other than pathogen biology, no one value can be used across countries and settings. Estimates in the literature of rubella’s R_0_ range from 2 (Edmunds et al. 2000) to 12 (Cutts et al. 2000) [3], [4]. In addition, R_0_ is generally measured indirectly; hence there may be substantial uncertainty as to its value, even in a particular setting. This puts public health officials in a quandary. The two pieces of the puzzle they know, birthrate and vaccine coverage, are useless without knowing a value whose measurement requires time, resources and expertise. For instance, in a country with a birthrate of 33 per 1,000 and 60% vaccine coverage, introduction of rubella vaccine will decrease CRS cases if R_0_ is 6.8 or lower, while CRS will increase of R_0_ is greater than 6.8.

Here, we present an analysis aimed at helping policy makers, program funders and other stakeholders reason about the utility of introducing rubella vaccination in specific settings while taking into account the uncertainty in the underlying transmission dynamics of the disease. We develop a framework for presenting our results that aims to be intuitive and easy to use by a non-technical audience, while not obscuring the technical details from those who are interested. This approach may also serve as a basis for decision making in other settings where substantial uncertainty exists.

## Methods

### Determining R_0_ Thresholds for Rubella Introduction

For a given R_0_, birthrate, and vaccine coverage we simulated 30 years of rubella incidence using a previously described age structured TSIR model [5], and determined whether the number of CRS cases increased or decreased when compared to having the same R_0_ and birthrate but no vaccination. The model assumes mild seasonal forcing of transmission [6], and that vaccine efficacy reaches a maximum of 0.97 [7]; various structures of contact between age classes were deployed, including constant, and the empirically derived POLYMOD structure [8].

For any particular birthrate and vaccination level, there exists a critical threshold of R_0_. If the true value of R_0_ is below this threshold, then the number of cases of CRS will decrease if vaccination is introduced with the specified coverage; while if the true value of R_0_ is above this threshold, then vaccination will lead to an increase in cases. To determine this threshold for each birthrate and vaccine coverage, we performed a binary search of possible values of R_0_ [9], starting at 20 and terminating when we had narrowed the search when CRS cases varied by at most ±1 case over 30 years relative to no vaccination (at the true threshold value CRS cases are the same with and without vaccination).

### Estimation the Distribution R_0_s

We estimated R_0_ based on the age distribution of infection using laboratory-confirmed rubella case data collected as part of WHO measles surveillance in 40 different countries in Africa from 2002–2009 (from Table 1 in Goodson et at al. 2011 [10]). Rubella is thought to be endemic throughout Africa. Relatively little detail is available on the epidemiology of rubella on the continent, though it appears the countries have primarily annual epidemics (see Goodson et al., 2011 [10]). None of the countries considered here had introduced rubella vaccine during the period considered, hence the age distribution of cases can be used to provide an estimate or R_0_.

Age specific rubella case reports were grouped into 5 age classes: less than 1 year of age, 1–4 years of age, 5–9 years of age, 10–14 years of age and 15 or more years of age.

For each country 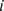, the log basic reproductive number is assumed to come from a normal distribution with mean 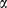 and variance 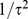:
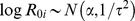



The force of infection, 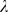, assumed to be constant over a person’s lifetime, is related to 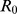 as:
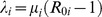



Where 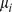 is the birthrate for country 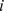 [11]. The likelihood of age specific case reports are calculated assuming a constant hazard of infection beginning at 9 months of age (i.e., that children are protected from rubella by maternal antibodies before reaching 9 months of age).

Parameters (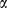 and 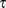) and country specific values of R_0_ were estimated using Bayesian Markov Chain Monte Carlo (MCMC) methods with non-informative priors (two chains 1,000,000 iterations, 500,000 iteration burn in). Convergence was assessed by visual examination of chains and posterior distributions and an 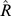 statistic of less than 1.01 [12]. This expected distribution of R_0_s in a random country was determined by integrating 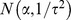 over the posterior distribution of parameters using MCMC methods.

### Figure Design

The data on the distribution of R_0_s and the threshold value of R_0_ are combined to make a figure summarizing our confidence that rubella vaccination would result in a reduction of CRS cases. The figure is a grid, where each cell represents a particular combination of birthrate (indicated by the column) and vaccine coverage (indicated by the row). In each cell we print the R_0_ threshold value calculated as described above. The cell is shaded to reflect our confidence that the true R_0_ is below the threshold given the estimate distribution of R_0_s. That is, our confidence that CRS will decrease if the vaccine is introduced. Each cell is colored on a gradient from red to yellow to green, where red designates a high confidence that CRS cases would increase if rubella vaccine were introduced, yellowing shades represent decreasing confidence in an increase in CRS, and green represents 95% confidence that CRS cases would decrease if a vaccine were introduced.

### Scenarios

We considered scenarios where there was only routine rubella vaccination among children and infants, administered as part of a countries measles vaccination program, and where rubella vaccine was administered in combination with supplemental immunization activities (SIAs). We considered SIAs with 60% coverage conducted every 4 years targeting 1–4 year olds, and every 4 years SIAs combine with a kickoff campaign in 1–14 year olds conducted in the first year of rubella vaccination.

We considered two different scenarios of population mixing. In the first we assume that age groups mix evenly, and individuals are no more likely to be infected by a member of a different age group than their own. In the second, we assume that there is assortative mixing and differences in the frequency of infectious contact by age. We assume that assortativity and contact frequency are proportional to what was measured in POLYMOD [8], a study of potentially infectious contacts conducted in 8 European countries.

All statistical analyses were done using R 2.15 (www.r-project.org).

## Results

**Figure 1:**
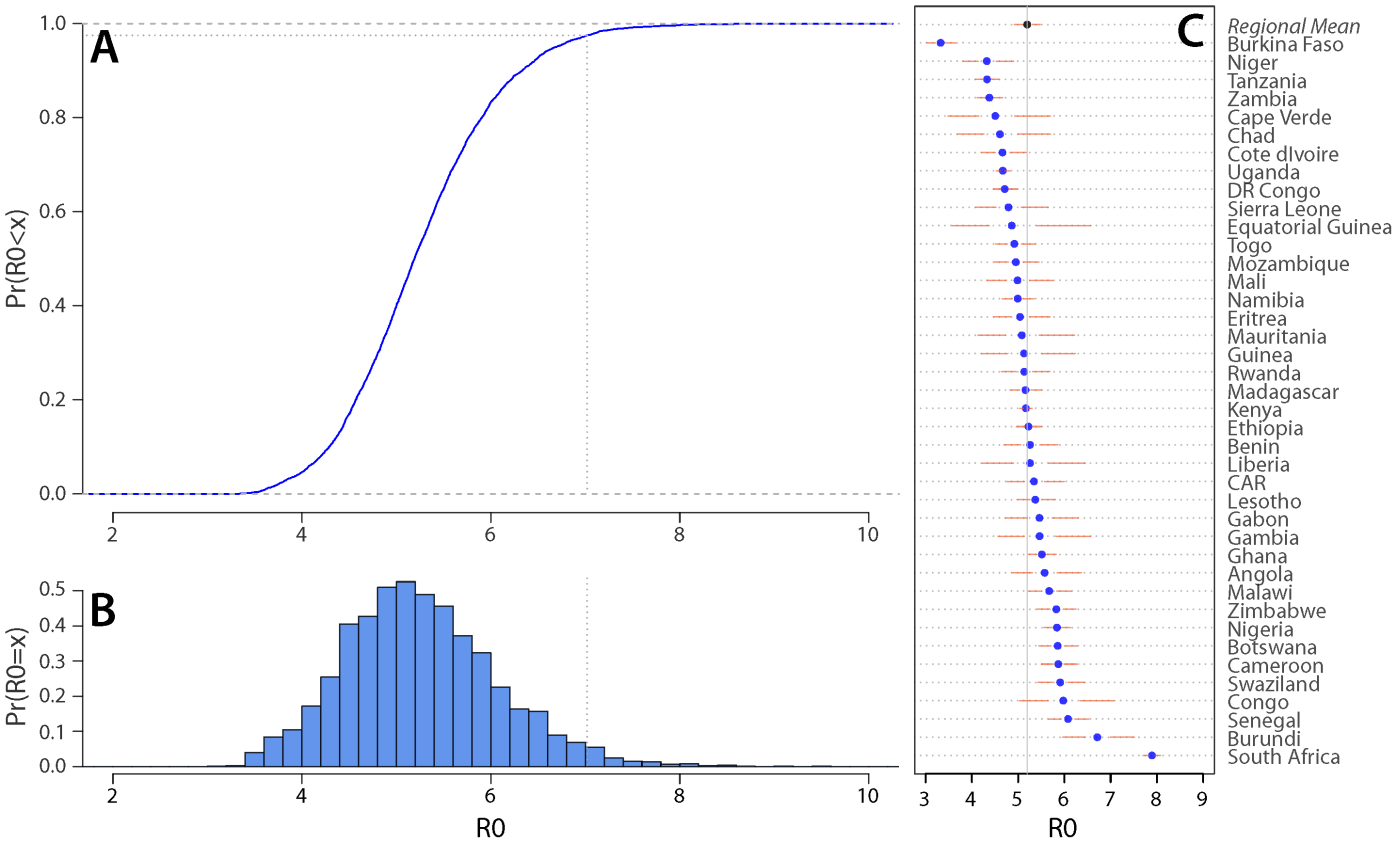
Estimated R_0_ distribution. (A) Cumulative distribution function, the dotted line represents 95^th^ percentile. (B) Probability distribution. (C) Individual country estimates; points indicate point estimates, gaps between points and solid lines the inter quartile range, and the range of the solid lines indicates the 95% credible interval for each country.

The median of the estimated R_0_ distribution for rubella is 5.2 (Figure 1C). If this distribution is taken to represent our confidence that the true value of R_0_ will be a particular value in a given setting, we are 90% confident that R_0_ will be between 4.0 and 6.7, and 50% confident will be between 4.7 and 5.7 (Figure 1A, 1B). Individual country estimates ranged from 3.3 (95% CrI: 3.0, 3.7) for Burkina Faso to 7.9 (95% CrI: 7.7, 8.1) for South Africa (Figure 1C).

**Figure 2:**
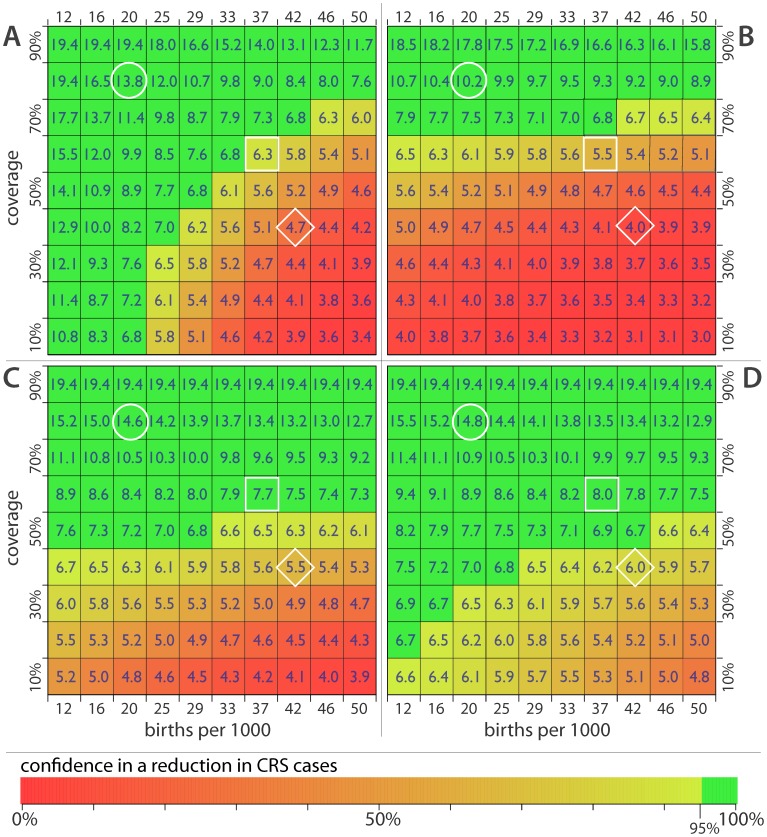
Critical R_0_ thresholds and confidence in seeing a reduction in CRS incidence for birthrate/vaccine coverage combinations. (A) Routine vaccination only, assuming even mixing across all population age groups. (B) Routine vaccination only, assuming assortative mixing and heterogeneities in contact between age groups. (C) Routine vaccination supplemented with SIAs of 1–4 year olds with 60% coverage every 4 years (assortative mixing). (D) Routine vaccinations and SIAs supplemented with a catch-up campaign covering 1–14 year olds with 60% coverage conducted when rubella vaccine is introduced. White circle shows the cell most closely corresponding to Guinea-Bissau. The square shows the cell most closely corresponding to Guinea Bissau. The diamond indicates the cell most closely corresponding to Somalia.

If only routine vaccination is used, countries with vaccine coverage greater than 80% can be highly confident in a reduction in CRS if they introduce rubella vaccine, while those with vaccine coverage less than 40% and a birthrate of 37 per 1,000 or higher are likely to see an increase in CRS cases if they introduce rubella vaccine (Figure 2). Particularly in low birthrate settings, the anticipated effect of introducing rubella vaccine is highly dependent upon our assumptions about mixing between age groups. Under more realistic mixing assumptions based on contact studies conducted in Europe, we predict that vaccination levels below 40% will result in an increase in CRS cases even in relatively low birthrate settings. In the following analyses we use these more realistic assumptions.

When routine rubella vaccination is supplemented by SIAs with 60% coverage conducted every four years, starting the year of vaccine introduction, we are confident in a decrease in CRS cases when routine vaccination levels are 60% or higher. Even if routine vaccination is as low as 50%, we are unlikely to see an increase in CRS cases from the introduction of the rubella vaccine. However, populations with low coverage and a high birthrate remain in the zone where an increase in CRS is likely. Substantial additional benefits can be realized by kicking off a rubella vaccination program with a large catch-up campaign covering 1–14 year olds at 60% coverage. In this scenario, only in the settings combining the highest birthrates with the lowest vaccination rates is an increase in CRS cases is likely.

To see how these results play out in real world situations, consider three countries with very different demographics and measles vaccine coverage: Nepal, Guinea-Bissau and Somalia (Figure 2). In 2010 the birthrate in Nepal was 22 per 1,000 and measles vaccine coverage was reported to be 86% (the circle in Figure 2). Here introduction of rubella vaccination into the routine program seems likely to result in a reduction in CRS cases, particularly if paired with SIAs. Guinea-Bissau (the square in Figure 2) had both a higher birthrate and lower vaccination coverage in 2010 (38 per 1,000, 61%) and a broad catch-up campaign followed by regular SIAs would be needed to safely introduce rubella vaccine. In a high birthrate, low vaccination setting, like Somalia (diamond in Figure 2, birthrate 43 per 1,000 and routine measles coverage of 46% in 2011), introducing rubella vaccine carry with it some risk even when paired with most aggressive SIA programs, and should only be considered after substantial improvements in routine vaccination or a major demographic change.

## Discussion

Overall, our results support the WHO recommendation that countries introduce rubella vaccine into their regular vaccination program if they can maintain coverage above 80% through a combination of routine vaccination and SIAs [13]. Under realistic assumptions about mixing between age groups the number of settings where we expect an increase in CRS goes up, however the 80% rule still seems adequate to avoid an increase in CRS. Using the charts presented here stake holders can get a sense of the likely outcome of adding rubella vaccine to a countries program without performing a sophisticated country specific modeling exercise, which may be time consuming and require extensive additional data collection. As more data on rubella incidence becomes available, these estimates can be updated to reflect our growing knowledge of rubella transmission dynamics.

**Figure 3:**
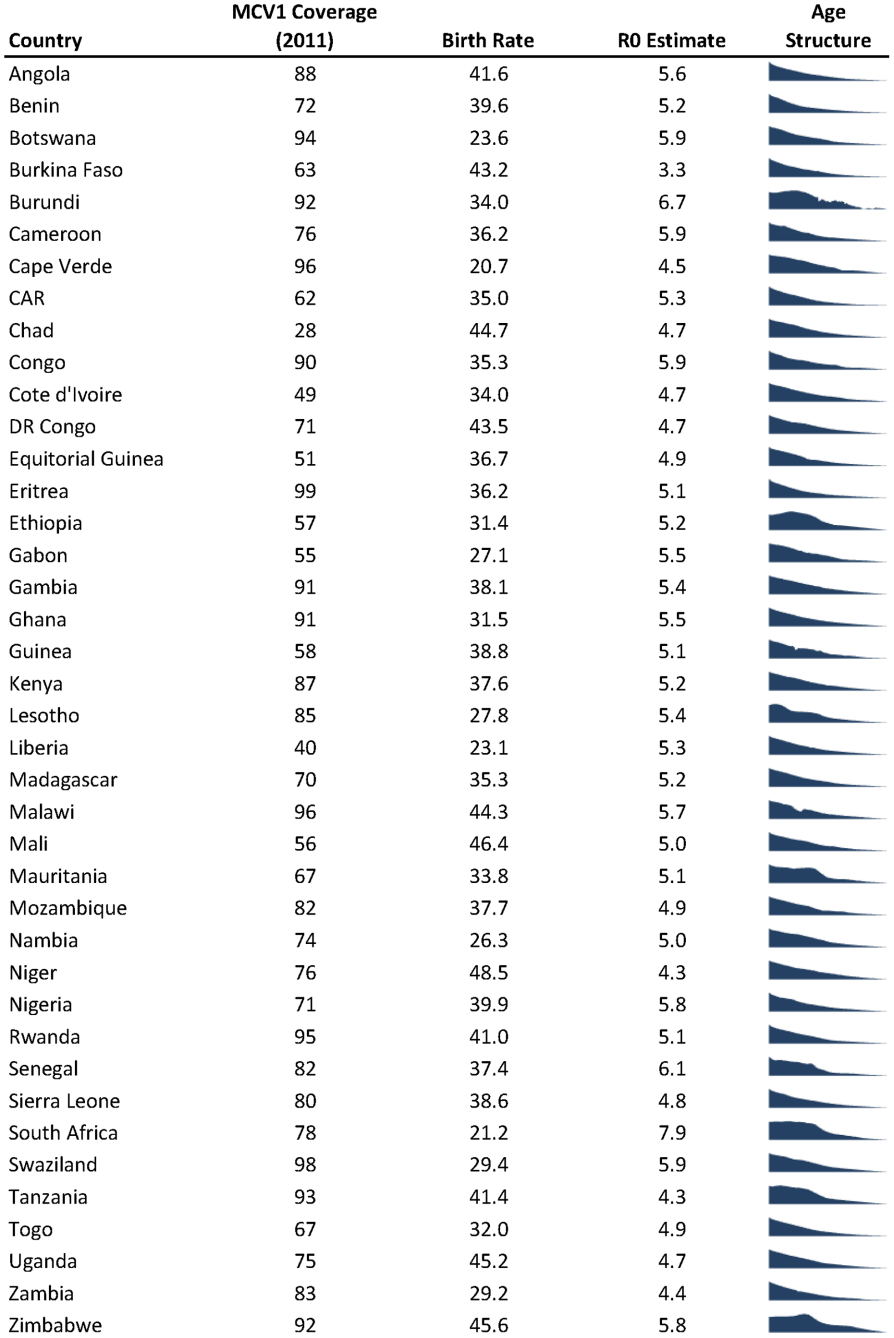
WHO-UNICEF estimated first dose measles (MCV1) vaccination coverage (from [27]) for 2011, 2010 birth rate (from [28]), R0 point estimate and population age structure (0–80 years of age, from [29]) for 40 African countries used in the analysis.

Of the 40 countries included in our R_0_ estimation, 19 had routine MCV vaccination rates of 80% or greater, and would be able to introduce rubella vaccination by the WHO criteria with no supplemental campaigns (Figure 3). Cape Verde, the only country considered here to have introduced rubella vaccine as of 2011 (vaccine was introduced in 2010) [14], has both high vaccination coverage and a low birthrate, hence is firmly in the “safe-zone”. Over the last year, Rwanda has added rubella to their immunization schedule, based on a funding window opened by GAVI; and Ghana and Senegal are expected to follow shortly [15]. While all three have high enough MCV1 coverage to justify introducing rubella vaccine, they have relative high birth-rates as well and should be careful to maintain high coverage.

By attempting to be general across a wide range of countries and settings, this work necessarily makes many generalizations and has several limitations. Data on rubella incidence is based on the analysis of suspected measles cases, and relies on surveillance systems that differ markedly by country and may be biased towards detecting rubella in particular age groups. Estimating the age distribution of rubella cases from measles surveillance data may bias the estimated age of infection downwards. However, this will tend to increase the estimate of R_0_, thereby increasing the predicted required coverage and resulting in more conservative predictions. Likewise, while most countries considered in this analysis have a pyramidal age structure, some do not (Figure 3); which may lead to a slight overestimation in the overall distribution of R_0_ across countries. While differences in age structure may affect estimates of R_0_, the critical threshold appears to be insensitive to drivers of age structure other than birth (i.e., mortality [16]) Assumptions about age specific mixing are based upon studies conducted in Europe, where MMR vaccine is already used widely, and may not apply to African and Asian countries considering introducing MR vaccine. However, the observation of assortative mixing by age has been replicated in many settings [17]–[19].

Previous work has, for the most part, either ignored the possible effect of assortative mixing between age groups on the introduction of rubella vaccine [20], or used simple matrix structures that many not fully capture increased mixing between parents and children [3], [21]. If assortative mixing is ignored, one would conclude that in low birth rate countries any level of rubella vaccination would lead to a reduction in CRS cases. However, under more realistic assumptions about how ages interact, low levels of vaccine coverage can lead to an increase in the incidence of CRS even when the birthrate is low. This is particularly troubling as increasing numbers of individuals in the United States and Europe decide to forgo MMR vaccination, dramatically decreasing coverage levels in some areas. Vaccine refusal tends to cluster geographically [22], and has already led to outbreaks of several previously eliminated childhood infections (e.g., measles, pertussis [22], rubella [23]). If this trend continues we may see a resurgence in CRS cases in developed countries.

Even if a country is within the range where introducing rubella vaccine is predicted to result in a reduction in CRS, countries must carefully consider their individual situation. Administrative coverage estimates may overestimate actual vaccine coverage [24], whether by underestimating the size of the target population or overestimating the number vaccinated. More importantly, if countries are achieving the coverage necessary to safely introduce rubella vaccine by supplementing routine coverage with SIAs, it is imperative they keep performing regular SIAs until their routine vaccination program achieves sufficient coverage. Regardless of how coverage is achieved, if levels of rubella vaccination drop off then CRS cases may increase. Conversely, vaccination in the private sector may increase the risk of CRS and change the risk-benefit tradeoff with the introduction of vaccine through state sponsored programs.

The threshold R_0_ identified for each birth rate/vaccination coverage combination is such that vaccination reduces the cumulative burden of CRS over 30 years, but makes no predictions about transient increases in the CRS burden. Previous reports of CRS increases in Greece and Costa Rica likely reflect this pattern [1], [2], [25], with single years where a large number of CRS cases occur, despite an overall decline in cumulative CRS burden. Policy makers should consider whether such events can be dealt with by the health system and the degree to which such events may adversely affect attitudes towards vaccination.

Our projections also ignore the effect of local disease dynamics on CRS burden. Local extinction of rubella may lead to an increase in the CRS burden by allowing individuals to age into childbearing years without exposure to the infection, up until the point where rubella is re-introduced [6], [16]. Local extinction may be particularly likely in areas where population mixing of more remote communities with central population centers is rare. Analysis of detailed data from South Africa makes it clear such extinctions regularly occur in that country [16], but how widespread this is and the interaction with birth rates remains unclear. Heterogeneity in vaccination coverage then becomes an important issue, as the CRS burden may increase in under-served districts. This is particularly likely to be an issue when vaccination policy is defined at a scale that differs from that of transmission [26]; and should be another consideration for policy makers.

Rubella vaccination is part of a renewed focus on vaccination through *Decade of Vaccines* and other initiatives by the funding community and public health agencies. Like the other vaccines in these initiatives, rubella vaccine has the potential save lives and prevent serious morbidity. However, unlike many vaccines, the introduction of rubella vaccines carries some risk. Because of uncertainties in rubella epidemiology, the case for vaccine introduction will not always be cut and dry. The charts and methods presented here aim to help funders, policy makers and other stakeholders make decisions about rubella vaccination while accounting for this uncertainty. These decisions can be made easier by continued research into the epidemiology of rubella, alternate approaches to predicting changes in CRS risk and, critically, careful monitoring of CRS incidence after the introduction of rubella vaccine.
